# Impact of Cooking on the Antioxidant and Functional Properties of Milling Fractions from Cold-Adapted Dutch Sorghum

**DOI:** 10.3390/foods15203672

**Published:** 2026-10-16

**Authors:** Francis Ala Adjetey, Daniela D. Herrera-Balandrano, Daniel Zogona, Hamza Mameri, Trust Beta

**Affiliations:** 1 Department of Food and Human Nutritional Sciences, University of Manitoba, Winnipeg, MB R3T 2N2, Canada; adjeteyf@myumanitoba.ca (F.A.A.); daniela.herrerabalandrano@umanitoba.ca (D.D.H.-B.); daniel.zogona@umanitoba.ca (D.Z.); 2 Ingénierie des Agropolymères et Technologies Émergentes (IATE), Université de Montpellier, INRAE, L’Institut Agro Montpellier, F-34060 Montpellier, France; hamza.mameri@inrae.fr

**Keywords:** sorghum bicolor, cold-adapted genotypes, bioactive compounds, cellular antioxidant activity, thermal processing

## Abstract

Sorghum is increasingly recognized for its nutritional and functional benefits, yet its application in temperate regions remains limited. This study examined the effects of cooking at 100 °C for 30 min on total phenolic content (TPC), total anthocyanin content (TAC), and antioxidant activity across whole grain, decorticated grain, and bran fractions from four cold-adapted Dutch sorghum genotypes (Dusormil HD7, HD19, HD100, and HD101). Black-grained genotypes (HD100 and HD101) exhibited the highest TPC and TAC, with uncooked HD100 bran reaching 39.49 mg FAE/g (TPC) and 151.10 mg LUE/L (TAC). Cooking led to a 64% increase in TPC in HD19 bran, while decorticated samples showed TPC reductions up to 70%. TAC consistently decreased post-cooking, with declines up to 39% in HD100 bran and 30% in whole grain. Antioxidant capacity, as measured by ABTS, decreased in most fractions after cooking, notably by 28% in HD100 bran from 1175.49 ± 24.2 mg TE/g to 845.81 ± 44.13 mg TE/g. HPLC analysis tentatively identified luteolinidin and apigeninidin as the predominant anthocyanins, with concentrations generally decreasing after cooking and being highest in bran. Cellular antioxidant activity (CAA) assays revealed that bran and decorticated samples, particularly HD19 at 400 μg/mL (42.91 ± 2.66 Units), maintained the highest post-cooking values, while the lowest was observed in decorticated HD100 at 200 μg/mL (5.54 ± 0.50 Units). These results emphasize the critical influence of genotype and processing on sorghum phytochemical retention, supporting the development of functional foods for temperate climates.

## 1. Introduction

The rising prevalence of non-communicable diseases has increased interest in plant-based whole foods, particularly grains, fruits, and vegetables [1]. These foods are highly valued for their rich profile of essential nutrients, minerals, dietary fiber, and bioactive phytochemicals that collectively improve cellular health and the general well-being of regular consumers [2]. Among these functional foods, sorghum ranks among the world’s major cereals in terms of planted area and harvested metric tons, following rice, wheat, maize, and barley, making it a crop of significant importance [3,4,5]. The inherent resilience of sorghum to adverse environmental conditions, coupled with its potential to address food security challenges in the context of accelerating population growth, has positioned this ancient crop as a critical component of sustainable agricultural systems worldwide [6,7,8]. In addition to its nutritional value, sorghum has significant industrial potential as a gluten-free flour and functional ingredient in processed foods. Its ability to grow under suboptimal or low-fertility conditions further increases its attractiveness as a sustainable raw material [2,9].

Sorghum is recognized for its high concentration in phenolic compounds such as phenolic acids, tannins, and flavonoids, as well as other phytochemicals such as polyamines [10,11]. These diverse bioactive compounds collectively contribute to sorghum’s well-documented health benefits, encompassing cholesterol reduction, anti-cancer properties, potent antioxidant capacity, and significant anti-inflammatory effects [2,7,9]. The 3-deoxyanthocyanidins (3-DXAs) present in pigmented sorghum varieties are particularly noteworthy, as they exhibit enhanced thermal and chemical stability compared to conventional anthocyanins, rendering them valuable candidates for functional food applications [12]. However, the bioaccessibility and ultimate bioavailability of these bioactive compounds are substantially affected by various factors, most critically including the application of different processing techniques [13,14]. Understanding how processing, particularly thermal treatments, influences the stability, extractability, and biological availability of these compounds is essential for optimizing the nutritional value of sorghum-based foods [15]. Common sorghum processing methods include boiling, steaming, baking, and extrusion, each influencing phytochemical retention and antioxidant activity through differences in processing parameters and matrix effects. These variations have important implications for both industrial applications and consumer health [15,16].

Diverse sorghum varieties and genetic accessions are conventionally classified according to their bran color phenotypes, including white, red, brown, and lemon-yellow varieties, with color variation primarily influenced by complex interactions between genotype, environmental conditions, and agronomic management practices [17]. While darker-pigmented variants, particularly those with black or deep red pericarps, typically accumulate higher concentrations of phenolic compounds and condensed tannins, lighter-colored varieties often display greater flavonoid diversity, with lemon-yellow cultivars frequently showing the highest concentrations of certain flavonoid classes [18,19]. In the context of cold-adapted genotypes specifically developed for European climates, previous characterization has shown that these Dutch sorghums (HD7, HD19, HD100 and HD101) possess unique starch granule morphologies and thermal properties, with gelatinization temperatures ranging between 61.86 °C and 77.44 °C [20]. Decorticated grain fractions, produced through mechanical bran removal, are valued for their reduced tannins and improved palatability, making them suitable for refined flours and processed foods. However, decortication may decrease phytochemical content, thereby reducing nutritional quality and bioactivity. Nevertheless, decorticated sorghum can support product diversification and expand market opportunities for gluten-sensitive consumers [17,21].

Because sorghum is rarely consumed raw, understanding how cooking affects the stability and antioxidant capacity of bioactive compounds in different milling fractions is essential. However, the effects of cooking on total phenolic content (TPC), total anthocyanin content (TAC), individual 3-deoxyanthocyanidins (3-DXAs), and antioxidant activity across whole-grain, decorticated grain, and bran fractions of cold-adapted Dutch sorghum genotypes remain poorly understood. A limitation of the present study is that it does not assess the in vivo bioavailability or direct health effects of these phytochemicals. This study evaluates the quantitative and qualitative impacts of cooking on TPC, TAC, and 3-DXAs in cold-adapted sorghum genotypes from European production systems, as well as their consequent antioxidant potential as determined through multiple in vitro and cellular assays. These findings may assist consumers and the food industry in making informed dietary and formulation decisions and support the development of value-added sorghum-based food products. To the best of our knowledge, limited information is available on the impact of cooking on phenolic compounds, 3-DXAs, and antioxidant functionality of cold-climate-adapted Dutch sorghum genotypes developed for production in temperate regions.

## 2. Materials and Methods

### 2.1. Sample Preparation

**Figure 1:**
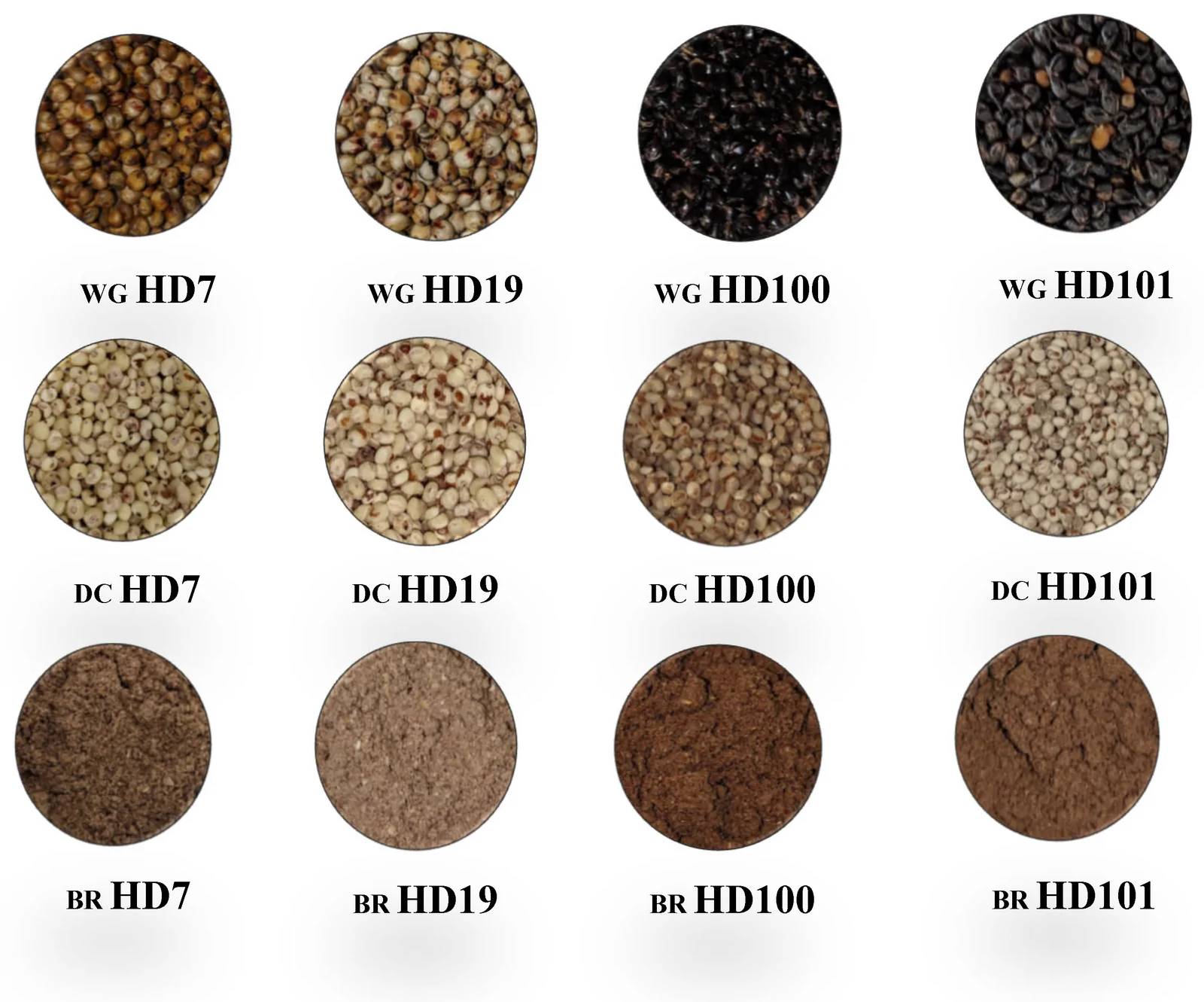
Images of Dusormil sorghum genotypes, along with their respective decorticated and bran fractions. WG, whole grain; DC, decorticated; BR, bran.

Cold-adapted sorghum genotypes, developed under the Dusormil breeding program, were obtained from Maatschap de Milliano-Meijer (Oostburg, Netherlands) and cultivated at 51° Northern Latitude 3° Eastern Latitude under cold climate conditions [20]. The grains included two genetic variations of *Sorghum nigricans*: Dusormil HD100 and HD101, and two *Sorghum bicolor* varieties: Dusormil HD7 (brown-grained) and Dusormil HD19 (white-grained) (Figure 1). Each genotype was divided into whole grain, bran and decorticated (endosperm) fractions. All sorghum varieties were decorticated following the method described by Lestienne et al. [21]. Briefly, the grains were steeped in water in sealed Erlenmeyer flasks and incubated in a water-bath shaker (VWR Digital Water Bath, Radnor, PA, USA) at 20 °C for 16 h. Thereafter, an abrasive decortication was performed on 80 g of sorghum grains a laboratory huller (TM 050, Satake, Stockport, UK) at 800 rpm, removing the outer layers. Approximately 15% of the initial grain weight was removed as bran (outer grain layers), resulting in separate bran and decorticated grain fractions.

### 2.2. Sample Cooking

Whole grains and milling fractions were cooked in a water at 100 °C for 30 min with a 1:6 sample-to-water ratio, with stirring every 5 min, using a Fisherbrand Isotemp digital hotplate stirrer (Thermo Fisher Scientific, Shanghai, China). Post-cooking samples were cooled, freeze-dried for 62 h using a Freeze-dryer (Virtis Genesis 25L, Warminster, PA, USA), and milled to a fine powder using a ball mill MM400 (Retsch, Hann, Germany) under liquid nitrogen (25 Hz for 4 min). Before analysis, samples were passed through a 0.5-mm sieve for homogenization.

### 2.3. Extraction of Phenolic Compounds

Phenolic compounds were extracted following a modified method of Apea-Bah et al. [7]. Briefly, 0.5 g of each powdered sample was mixed with 10 mL of 1% HCl in methanol and subjected to sonication for 1 h at room temperature using an ultrasonic cleaner (Branson 5510R-DHT, CT, USA), then centrifuged at 7500× *g* for 10 min using a Sorvall RC6 Plus centrifuge (Thermo Fisher Scientific, Wilmington, NC, USA). After centrifugation, the supernatant was collected in a 50 mL centrifuge tube and protected from light with aluminum foil. The extraction was performed twice, and the supernatants from the same sample were combined and concentrated using a rotary evaporator (RV 10 digital Rotary Evaporator, IKA, Staufen im Breisgau, Germany), then reconstituted in 5 mL of 50% aqueous methanol, and stored at –20 °C for subsequent analyses.

### 2.4. Evaluation of Total Phenolic Content (TPC)

TPC was evaluated following the Folin–Ciocalteu method, according to Apea-Bah et al. [22]. A standard curve was prepared with ferulic acid (0.025, 0.050, 0.100, 0.150, and 0.200 mg/mL). An aliquot (18.2 µL) of extracts or standard solutions was pipetted into a 96-well microplate, and 36.4 µL of Folin–Ciocalteu solution and 145.4 µL of sodium carbonate (Na_2_CO_3_) solution were added. The mixture was incubated in darkness at room temperature for 1 h. Absorbance was measured at 750 nm using a Biotek ELx800 microplate reader (ELx800, BioTek Instruments Inc., Winooski, VT, USA) set to 37 °C. TPC was expressed as mg ferulic acid equivalent per gram of sample (mg FAE/g) on a dry weight (DW) basis. Ferulic acid was selected as the calibration standard because it is the predominant phenolic acid in cereal grains and therefore provides a more representative estimate of TPC in sorghum samples.

### 2.5. Evaluation of Total Anthocyanin Content (TAC)

TAC was conducted in accordance with the pH-differential method described by Kumari et al. [23]. The extracts were aliquoted (0.2 mL) into two separate tubes. The first aliquot was mixed with 1.8 mL of pH 1.0 buffer (125 mL of 0.2 N KCl and 385 mL of 0.2 N HCl), while the second aliquot was diluted with 1.8 mL of pH 4.5 buffer (400 mL of 1 N sodium acetate, 240 mL of 1 N HCl, and 360 mL of distilled water). After 20 min of incubation in the dark at room temperature, the absorbance values were measured at 480 nm and 700 nm for both the pH 1.0- and pH 4.5-diluted aliquots using a Themo Scientific GENESYS 10S UV-VIS spectrophotometer (Waltham, MA, USA). Total monomeric anthocyanins were computed based on the disparity in absorbance readings between the two buffer systems using the formula below:

TAC (LUT equivalents mg/L) = (A × MW × DF × 10^3^)/ε × L

where A = (A_480_ − A_700_)_pH1_ − (A_480_ − A_700_)_pH4.5_, MW = molecular weight of Luteolinidin (LUT) (271.24 g/mol), DF = Dilution factor, L = path length in cm (1 cm), ε = molar extinction coefficient of Luteolinidin (31,700 L × mol × cm^−1^).

### 2.6. HPLC and Spectrophotometric Analyses

#### 2.6.1. UV-Visible Spectrophotometric Analysis

A UV-Vis spectrophotometer was used to determine the maximum absorption wavelengths of the sorghum extracts over a wavelength range of 320–750 nm. For each sample, the absorbance spectrum was recorded, and characteristic peaks were observed at approximately 480 nm and 460 nm, corresponding to luteolinidin (LUT) and apigeninidin (AP), respectively. The assignment of these peaks is consistent with previous reports by Awika et al. [24] and Kumari et al. [23], who identified absorption maxima for LUT and AP at 482 nm and 468 nm, respectively, under acidic conditions (pH 1).

#### 2.6.2. HPLC Analysis

The HPLC analysis was carried out using the method of Bellido & Beta [25]. Samples (20 µL) were analyzed using a Waters 2695 HPLC system (Waters, Milford, MA, USA) equipped with a 996-photodiode array detector. Anthocyanin separation was achieved on a Luna 3 µm C18 column (150 mm × 3 mm) maintained at 35 °C. The mobile phase consisted of 4.5% formic acid in deionized water (solvent A) and 100% methanol (solvent B) with the following gradient: 10% B at 0 min, 25% B at 30 min, 33% B at 34 min, 90% B at 42 min, returning to 10% B from 45 to 50 min. The flow rate was 0.4 mL/min, and the total run time 50 min. Detection was at 480 nm, and compounds were tentatively identified based on retention time and UV spectra in comparison with published data. Luteolinidin, apigeninidin, and peonidin were the primary anthocyanins evaluated, with peonidin consistently detected at low to moderate levels.

### 2.7. Evaluation of Antioxidant Capacity

#### 2.7.1. ABTS Assay

The ABTS assay was conducted using the protocol of Apea-Bah et al. [22]. A 16 mM ABTS solution was prepared and mixed with 6 mM potassium persulfate solution in a 1:1 ratio to generate the ABTS radical, which was then incubated at room temperature (~25 °C) for 16 h in the dark. The stock solution was appropriately diluted with 200 mM phosphate-buffered saline (PBS) to produce the ABTS working solution. A Trolox standard curve was prepared (50–800 µM). For the assay, 10 µL of sample, standard, or blank and 190 µL of ABTS working solution were pipetted into a 96-well microplate. After incubation at room temperature for 60 min in the dark, absorbance was measured at 750 nm on a microplate reader (ELx800, BioTek Instruments Inc., Winooski, VT, USA). Antioxidant activity was calculated from the Trolox standard curve. The ABTS was expressed as mg Trolox equivalents per gram of sample (mg TE/g DW).

#### 2.7.2. DPPH Assay

The DPPH radical scavenging activity was determined following the method of Apea-Bah et al. [22]. A 60 µM DPPH solution and a Trolox standard curve (50–800 µM) were prepared. For the assay, 5 µL of sample, standard, or blank and 195 µL of DPPH solution were added to each well of a 96-well microplate. The mixture was incubated for 60 min at room temperature in the dark. Absorbance was measured at 515 nm using a Biotek ELx800 microplate reader (Winooski, VT, USA) set to 37 °C. Antioxidant activity was calculated using the Trolox standard curve and expressed as mg Trolox equivalents per gram of sample (mg TE/g) DW).

#### 2.7.3. Cellular Antioxidant Activity (CAA)

The CAA of anthocyanin extracts of the cooked Dusormil sorghum samples was determined following the method reported by Wolfe & Rui [26]. Anthocyanin extracts of the cooked Dusormil samples were prepared at concentrations of 200 and 400 µg/mL in serum-free Dulbecco’s Modified Eagle Medium (DMEM). 2′,7′-Dichlorodihydrofluorescin diacetate (H_2_DCFDA) probe at 2.5 µM was prepared in ethanol and diluted in serum-free DMEM. The working solution of the H_2_DCFDA probe was protected from light exposure. An oxidizing agent, 2,2′-azobis (2-amidinopropane) dihydrochloride (AAPH), was dissolved in phosphate-buffered saline (PBS) and diluted in serum-free DMEM to a final concentration of 200 µM.

The assay was conducted in a black 96-well microplate with a clear bottom, where Caco-2 cells were seeded at a density of 5 × 10^4^ cells in 100 μL of growth medium/well, and placed in an incubator (Thermo Scientific Steri-Cycle 370, Thermo Scientific, Marietta, OH, USA) at 37 °C in a humidified atmosphere containing 5% CO_2_ for 24 h. After incubation, the medium was removed and cells were washed with 100 µL of PBS. Subsequently, 50 µL of 2.5 µM H_2_DCFDA solution and 50 µL of cooked sorghum extracts were added to appropriate wells. The cells were incubated at 37 °C for 1 h. After incubation, the treatment solutions were carefully aspirated, and the cells were gently washed once with 100 µL PBS. Then 100 µL of 200 µM AAPH solution was added to each well. Cells treated with H_2_DCFDA and AAPH without sample extracts were set as the control.

The microplate was immediately transferred to a microplate reader (BioTek, Winooski, VT, USA) preheated to 37 °C, and fluorescence was monitored kinetically (excitation: 485 nm; emission: 538 nm). Fluorescence intensity was recorded every 5 min for a total of 60 min. The assay was performed in triplicate for each sample concentration. The area under the fluorescence curve (AUC) was calculated from the relative fluorescence unit (RFU) values obtained for each sample. CAA values were then calculated using the following equation based on the corresponding AUC values:

$$\mathrm{CAA}\;\mathrm{Units}=100-\frac{\mathrm{AUC}\;\mathrm{antioxidant}}{\mathrm{AUC}\;\mathrm{control}}\times 100$$

### 2.8. Statistical Analysis

All experiments were performed in triplicate. Statistical analyses were conducted using OriginPro software (version 2024, OriginLab Corporation, Northampton, MA, USA). Differences between means were assessed by one-way analysis of variance (ANOVA), followed by Tukey’s post hoc test for multiple comparisons at a 95% confidence level (*p* < 0.05). In addition, principal component analysis (PCA) was performed to visualize the clustering and relationships among sorghum genotypes and their respective fractions based on the measured variables.

## 3. Results and Discussion

### 3.1. Effect of Cooking on TPC in Sorghum Fractions

The distribution of TPC across the evaluated sorghum genotypes and their respective milling fractions is presented in Table 1. In whole grain fractions, TPC levels were significantly higher in the *S. nigricans* genotypes (HD100 and HD101) compared to the *S. bicolor* varieties (HD7 and HD19), a difference that was observed in both cooked and uncooked samples, indicating the stability of inherent genotype-specific phenolic profiles [3]. For instance, uncooked WG HD100 exhibited the highest TPC (15.83 ± 0.23 mg FAE/g), aligning with previously reported values for black-grained sorghum (5.3 to 19.8 mg/g GAE) [27]. Conversely, the decorticated endosperm fractions of all genotypes had the lowest TPC (1.35–2.59 mg FAE/g), suggesting that phenolic compounds are predominantly concentrated in the outer anatomical layers of the grain, such as the pericarp and seed coat [16].

Cooking had genotype- and fraction-dependent effects on TPC. Notably, whole grain and bran fractions of the *S. bicolor* varieties (HD7 and HD19) showed marked increases in extractable TPC following cooking. For example, TPC in HD19 whole grain increased by 63.75% after cooking (from 8.33 to 13.64 mg FAE/g, *p* < 0.05). Such increases likely result from the thermal disruption of cell wall matrix structures, which liberate otherwise bound phenolic acids, thus enhancing their extractability [28]. Similar, though smaller, increases were observed in the bran fraction of HD101 (6.68% post-cooking), while the bran of HD100 remained statistically unchanged, suggesting a high degree of thermal stability among the pigmented 3-DXAs in the *S. nigricans* genotypes [11].

Conversely, cooking consistently reduced TPC in the decorticated endosperm samples across all genotypes, with the most pronounced reductions in HD100 and HD7. This susceptibility to thermal degradation in endosperm tissues is likely linked to starch gelatinization, which alters granule organization and exposes phenolic compounds to oxidative degradation [29,30,31]. Previous work shows that these cold-adapted Dutch genotypes possess starch granules with relative crystallinity values of 24–31% and gelatinization temperatures (61.9–77.4 °C) below the 100 °C cooking temperature applied here [20], facilitating the complete thermal disruption of starch structures. This process may expose previously protected phenolic compounds to rapid, non-enzymatic oxidation and possible reaction with proteins [32,33]. Taken together, these results might indicate that while the bran layer provides a protective biochemical matrix that stabilizes and even enhances phenolic recovery during thermal processing, the starchy endosperm fundamentally lacks this protective structure, thereby leading to the possibly accelerated degradation of phenolic compounds under typical wet-heat conditions [34,35].

The distinct response to cooking among genotypes is particularly noteworthy. Although *S. nigricans* genotypes maintained the highest absolute TPC values, thermal processing allowed the *S. bicolor* varieties (HD7 and HD19) to significantly narrow the gap in TPC, particularly in the bran fractions. This suggests that the health-promoting potential of certain milling fractions, especially HD19 bran, may be underestimated if only analyzed in the raw state [35]. Overall, these results highlight the importance of evaluating the “as-consumed” nutritional profiles of sorghum, as cooking can selectively increase extractable phenolic content depending on the genotype and grain fraction [29].

### 3.2. Effect of Cooking on TAC in Sorghum Fractions

The distribution of TAC across sorghum genotypes and milling fractions is shown in Table 1. In uncooked fractions, *S. nigricans* genotypes (HD100 and HD101) exhibited higher TAC than *S. bicolor* varieties, with this difference most pronounced in the bran fractions. For example, uncooked HD100 bran reached the highest TAC, followed by HD101, reinforcing the view that anthocyanins are primarily localized in the outer grain layers, particularly in the pigmented glumes and pericarp of *S. nigricans* [16].

Cooking reduced TAC by up to 39% in HD100 bran and 30% in HD100 whole grain. For whole grain samples, a general decline in TAC was observed after cooking, with HD101 demonstrating the highest stability (only a 3.23% reduction). Notably, the bran fractions of HD7 and HD101 were highly resilient to cooking, exhibiting no significant change in TAC, indicating that certain cold-adapted genotypes produce bran anthocyanins with greater thermal stability than previously reported [34]. In contrast, the decorticated fractions of *S. nigricans* genotypes (HD100, HD101) were more susceptible to heat-induced anthocyanin loss.

Remarkably, decorticated fractions from *S. bicolor* genotypes (HD7 and HD19) showed a significant increase in extractable TAC following cooking, with HD19 exhibiting the greatest enhancement. As discussed above, the unique starch properties of these Dutch genotypes, including elevated gelatinization temperatures [20], may facilitate the formation of a porous gelatinized network, enhancing anthocyanin release and potentially reducing oxidative loss [33]. Thus, anthocyanin retention and extractability upon cooking are determined by both genotype and anatomical fraction.

Overall, these results indicate that the TAC in black *S. nigricans* genotypes are generally more susceptible to thermal processing than those in the white and brown *S. bicolor* varieties, but that select “as-consumed” fractions, such as cooked *S. bicolor* grains, may exhibit unexpectedly high antioxidant potential due to enhanced pigment extractability.

### 3.3. Identification and Relative Quantification of Anthocyanins

#### 3.3.1. UV-Visible Spectrophotometric Identification

**Figure 2:**
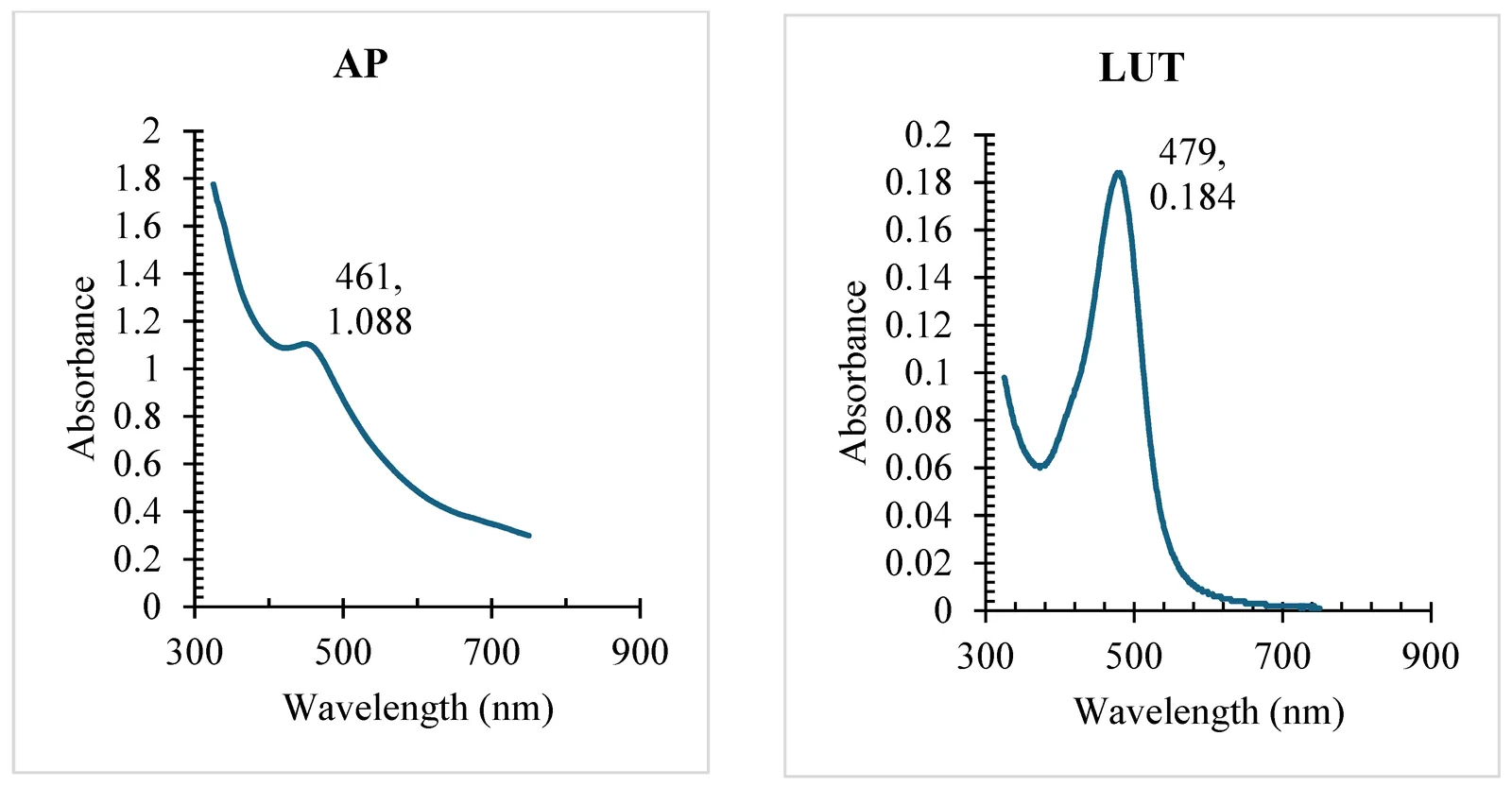
Maximum absorption wavelengths identified in sorghum extracts corresponding to the flavonoid compounds apigeninidin (AP) and luteolinidin (LUT).

UV-visible spectrophotometric analysis was used to determine the maximum absorption wavelengths characteristic of the sorghum extracts. Two distinctive primary absorption bands were identified at 480 nm and 460 nm consistently across the genotypes (Figure 2). These spectral features correspond to the predominant 3-DXAs found in sorghum, specifically luteolinidin (LUT) and apigeninidin (AP) [17]. Unlike conventional anthocyanins, 3-DXAs lack an oxygen atom at the C-3 position, a structural trait that imparts greater thermal and light stability [10]. This enhanced resilience makes 3-DXAs particularly valuable as natural colorants and bioactive compounds for functional food applications [12,36].

#### 3.3.2. Relative Quantification of 3-DXAs in Sorghum Fractions

**Figure 3:**
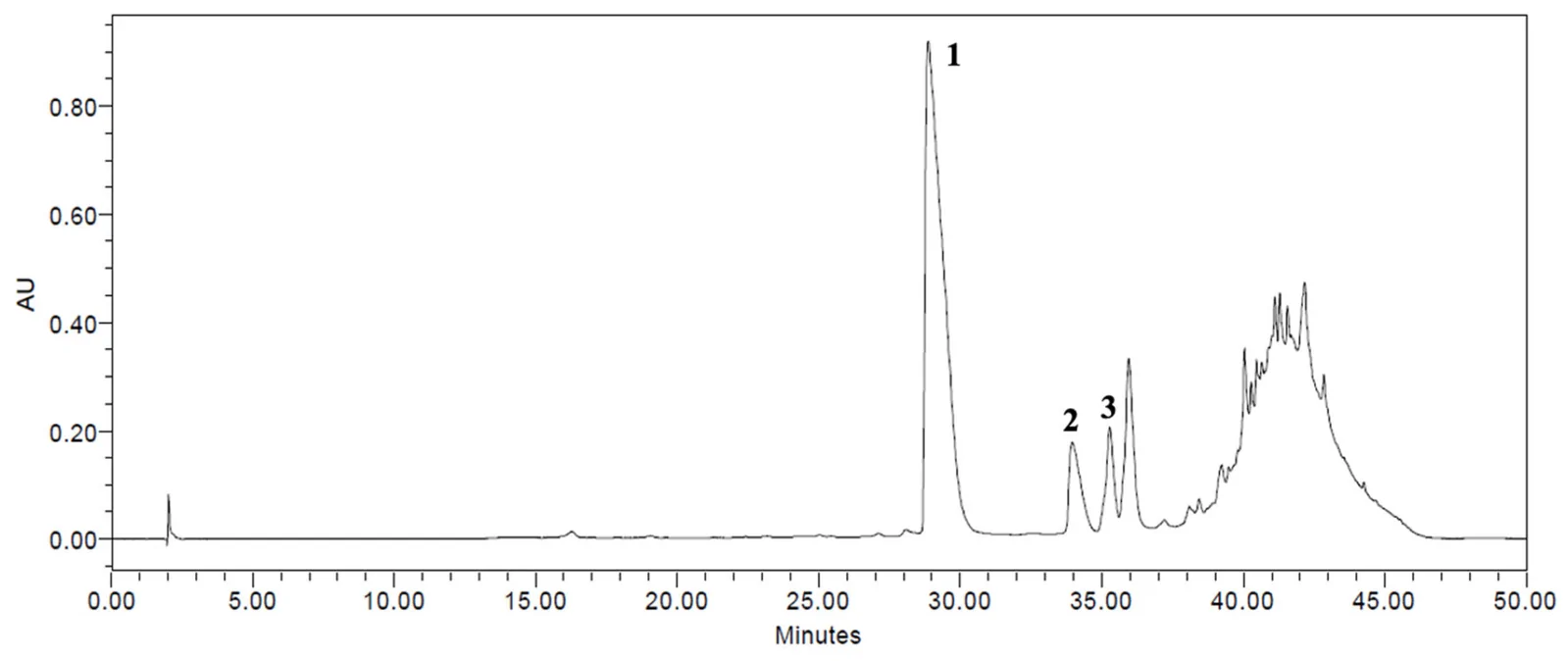
Representative HPLC chromatograms for all sorghum samples analyzed, detected at 480 nm. Peaks 1, 2 and 3 correspond to luteolinidin, apigeninidin and peonidin, respectively.

Figure 3 presents representative HPLC chromatograms of the analyzed sorghum grain fractions, demonstrating consistent elution patterns and retention times across samples. The major peaks correspond to luteolinidin (LUT), apigeninidin (AP), and peonidin, which were selected for quantification because of their prominence in sorghum and their recognized importance as major 3-DXAs in these genotypes [36]. These analytes were chosen based on their frequent identification in sorghum and their substantial contribution to the anthocyanin profile reported in previous studies.

High-performance liquid chromatography was then used to quantify the relative abundance of these 3-DXAs across the cooked and uncooked sorghum grain fractions, based on peak area integration (Table 2). The pigmented *S. nigricans* genotypes (HD100 and HD101) exhibited significantly higher levels of both LUT and AP compared to the lighter-colored *S. bicolor* varieties (HD7 and HD19), reflecting genotype-dependent differences in 3DXA accumulation [3]. In addition to luteolinidin and apigeninidin, peonidin was detected at low to moderate levels in most samples, with the highest concentrations found in bran fractions. While peonidin was generally less abundant than the primary 3-DXAs, it contributed noticeably to the anthocyanin profile in certain genotypes and processing conditions. These distribution patterns align with standard sorghum grain profiles observed in prior characterizations, such as those reported by Wu et al. [37].

Thermal processing had a fraction- and genotype-dependent impact on these 3-DXAs [12]. Luteolinidin generally displayed strong thermal stability across most grain fractions, whereas apigeninidin was more heat-labile, often declining significantly post-cooking. An exception was observed in the HD100 genotype, where apigeninidin levels in the cooked bran increased compared to the uncooked state, suggesting possible enhanced extractability or stabilization under certain matrix conditions. The remarkable persistence and conservation of 3DXA pigmentation observed in samples, particularly pronounced in the decorticated endosperm fractions following cooking, suggests a protective physicochemical relationship between the bioactive pigments and the overall grain matrix structure [12].

These observations may be related to previously reported starch properties of these genotypes, including elevated gelatinization temperatures and distinctive morphologies [20], which likely facilitate physical entrapment anthocyanins and offer protection against oxidative degradation [31,38]; however, direct evidence was not obtained in the present work. Thus, the observed thermal resilience of 3-DXAs might reflect a complex interplay between pigment chemistry and the dynamic structure of the grain matrix.

### 3.4. Evaluation of Antioxidant Activities of Cooked Sorghum Fractions

The antioxidant activities of the sorghum milling fractions, assessed by DPPH and ABTS radical scavenging assays, are presented in Table 3. Across all genotypes, the antioxidant capacity demonstrated a highly consistent gradient in decreasing potency following the order of bran > whole grain > decorticated endosperm. The highest radical scavenging activity values were observed in the bran fractions of the pigmented *S. nigricans* genotypes, with HD100 displaying the highest DPPH activity (180.20 ± 1.31 mg TE/g), closely followed by HD101 (173.30 ± 0.60 mg TE/g). Notably, the whole grain fractions across all four genotypes exhibited remarkably comparable DPPH radical scavenging potential, with values ranging narrowly from 161.82 to 166.42 mg TE/g. In contrast, the decorticated endosperm samples consistently had the lowest antioxidant activity values (~22–29 mg TE/g), underscoring the critical importance of the bran layer for the preservation and functionality of antioxidant compounds [34,38].

Thermal processing at 100 °C for 30 min demonstrated assay-dependent effects [15]. DPPH scavenging activity remained largely unchanged between uncooked and cooked samples, across most genotypes and fractions. However, the ABTS assay revealed greater sensitivity to cooking [28,39], with notable declines observed, particularly in the bran of HD100, where ABTS activity decreased from 1175.49 ± 24.2 mg TE/g to 845.81 ± 44.13 mg TE/g following cooking. This divergence suggests that different phenolic classes or antioxidant mechanisms are selectively influenced by thermal treatment [40]. Despite these selective losses, the overall antioxidant potential remained high in the bran and whole grain fractions, likely due to the protective physicochemical matrix provided by starch and protein networks in the grain [33,38].

Interestingly, these results contrast with previous reports [41], which indicated that red and brown sorghum pericarp extracts generally demonstrate substantially greater DPPH radical scavenging activity compared to black pigmented variants [23]. However, in our study, the cold-adapted black *S. nigricans* varieties (HD100 and HD101) consistently displayed equal or demonstrably superior antioxidant capacity compared to the lighter colored brown (HD7) and white (HD19) *S. bicolor* genotypes across multiple assay methodologies.

Overall, this genotype-specific distinction suggests that the specific pigmentation and phytochemical composition of these European-bred cold-adapted varieties differ notably from the antioxidant profiles typically documented for conventionally grown sorghum germplasm [19]. Additionally, the similarity between the whole-grain and bran fractions in the DPPH radical scavenging assay indicates that bioactive antioxidant compounds are substantially concentrated and highly accessible and potentially bioaccessible within the intact whole-grain matrix, rather than being restricted primarily to the bran layer [1]. These results highlight the potential nutritional and functional advantages of cold-adapted black sorghum for human consumption and food industry applications [42].

### 3.5. CAA of Cooked Sorghum Samples Against AAPH-Induced Oxidation

The antioxidant activity measured in vitro may not fully represent the actual biological antioxidant action within living organisms [43]. Chemical assays are frequently conducted under experimental conditions that differ from physiological environments. Moreover, Caco-2 cells represent an ideal in vitro model for assessing CAA and bioavailability, thus playing a crucial role in evaluating the biological activity of food antioxidants [44]. Therefore, we conducted the CAA assay, which is more physiologically relevant due to factors such as cellular uptake, metabolism, and intracellular distribution of antioxidant compounds [26].

**Figure 4:**
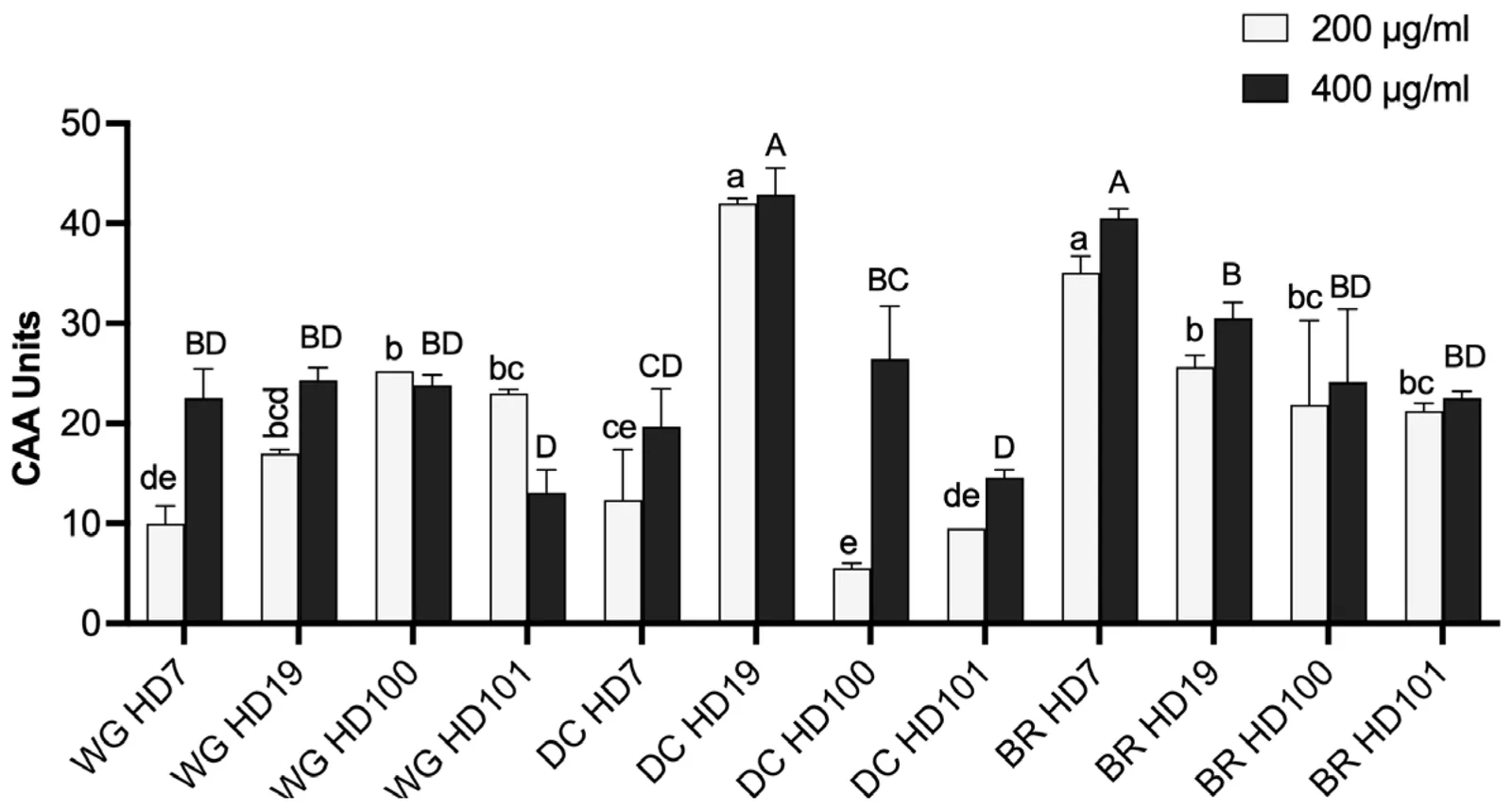
Cellular antioxidant activity (CAA) of cooked sorghum extracts. WG, whole grain; DC, decorticated; BR, bran. a–e, different lowercase letters represent significant differences among samples at the concentration of 200 µg/ml; A–D, different uppercase letters represent significant differences among samples at the concentration of 400 µg/ml, as determined by one-way ANOVA and Tukey’s multiple comparisons test.

The intracellular antioxidant activity of cooked Dusormil sorghum samples (whole grain, decorticated, and bran) at 200 and 400 µg/mL is presented in Figure 4. These concentrations were selected based on preliminary MTT assays, which demonstrated that raw and cooked whole-grain extracts at 200 and 400 µg/mL did not induce cytotoxicity in Caco-2 cells. Significant variations in CAA values were observed among the samples, ranging from 5.54 ± 0.50 Units (for DC HD100 at 200 µg/mL) to 42.91 ± 2.66 Units (for DC HD19 at 400 µg/mL). At the concentration of 200 µg/mL, DC HD19 exhibited the highest CAA (42.00 ± 0.49), followed by BR HD7 (35.09 ± 1.63), BR HD19 (25.66 ± 1.15), and WG HD100 (25.23 ± 0.00), while DC HD100 recorded the lowest CAA (5.54 ± 0.50 Units). WG HD101, BR HD100, and BR HD101 showed moderate antioxidant activities (23.02 ± 0.38, 21.84 ± 8.44, and 21.25 ± 0.78 Units, respectively). At the concentration of 400 µg/mL, DC HD19 still exhibited the highest CAA with 42.91 ± 2.66 Units, followed by BR HD7 (40.56 ± 0.94 Units), and BR HD19 (30.52 ± 1.63 Units), while DC HD101 and WG HD101 showed the lowest CAA values (14.62 ± 0.80 and 13.10 ± 2.29 Units, respectively).

Our results demonstrate that increasing the extract concentration to 400 µg/mL significantly enhanced the CAA for WG HD7, WG HD19, DC HD7, DC HD100, DC HD101, BR HD7, and BR HD19. In contrast, CAA decreased for WG HD100 and WG HD101, while remaining unchanged for DC HD19 and BR HD101. This variability suggests that concentration-dependent antioxidant activity might be influenced by the specific genotype, sample types, or the cellular uptake efficiency of bioactive compounds in the Dusormil sorghum extracts. In this study, the antioxidant activity of cooked Dusormil samples evaluated through DPPH and ABTS assays was consistently higher in the bran fractions of the four genotypes (HD7, HD19, HD100, and HD101) compared to their corresponding whole-grain and decorticated fractions. Despite the high TPC in HD100 and HD101, which is generally associated with strong antioxidant capacity [45], a reverse trend was observed in CAA. Specifically, the decorticated fractions DC HD100 and DC HD101 at 200 µg/mL presented the lowest CAA values, whereas DC HD19 showed the highest CAA at both concentrations. This discrepancy suggests that high TPC alone does not necessarily translate into greater intracellular antioxidant activity, as CAA depends not only on the abundance of antioxidant compounds but also on their cellular uptake, bioavailability, and interactions with cell membranes. The disparity between chemical antioxidant assays and CAA suggests that antioxidant effectiveness depends not only on phenolics but also on compound structure, cellular transport, intracellular localization, and metabolism. Our findings are consistent with those of Xiong et al. [46], who reported that antioxidant compounds’ interaction with cells is dependent on their ability to associate with cellular membranes and be internalized by the cells. The improved CAA observed indicates that Dusormil sorghum extracts could protect Caco-2 cells against AAPH-induced oxidation in a dose-dependent manner, with 400 µg/mL treatment counteracting ROS generation better than 200 µg/mL, except for WG HD100 and WG HD101.

Overall, our findings are aligned with recent metabolomics work on Dutch sorghum genotypes [47], which demonstrated significant effects of dehulling and cooking on phenolic distribution and bioaccessibility. While that study focused primarily on untargeted metabolomic profiling and protein digestibility, our approach provides complementary insight into anthocyanin stability, antioxidant activity, and cellular antioxidant responses across sorghum fractions and genotypes following thermal processing. Nevertheless, due to limited sample availability, cytotoxicity was not evaluated for the decorticated and bran fractions. All CAA assays were performed at concentrations previously confirmed to be non-cytotoxic in Caco-2 cells using whole-grain extracts. Consequently, this limitation should be taken into consideration when interpreting the CAA findings.

### 3.6. Principal Component Analysis (PCA)

**Figure 5:**
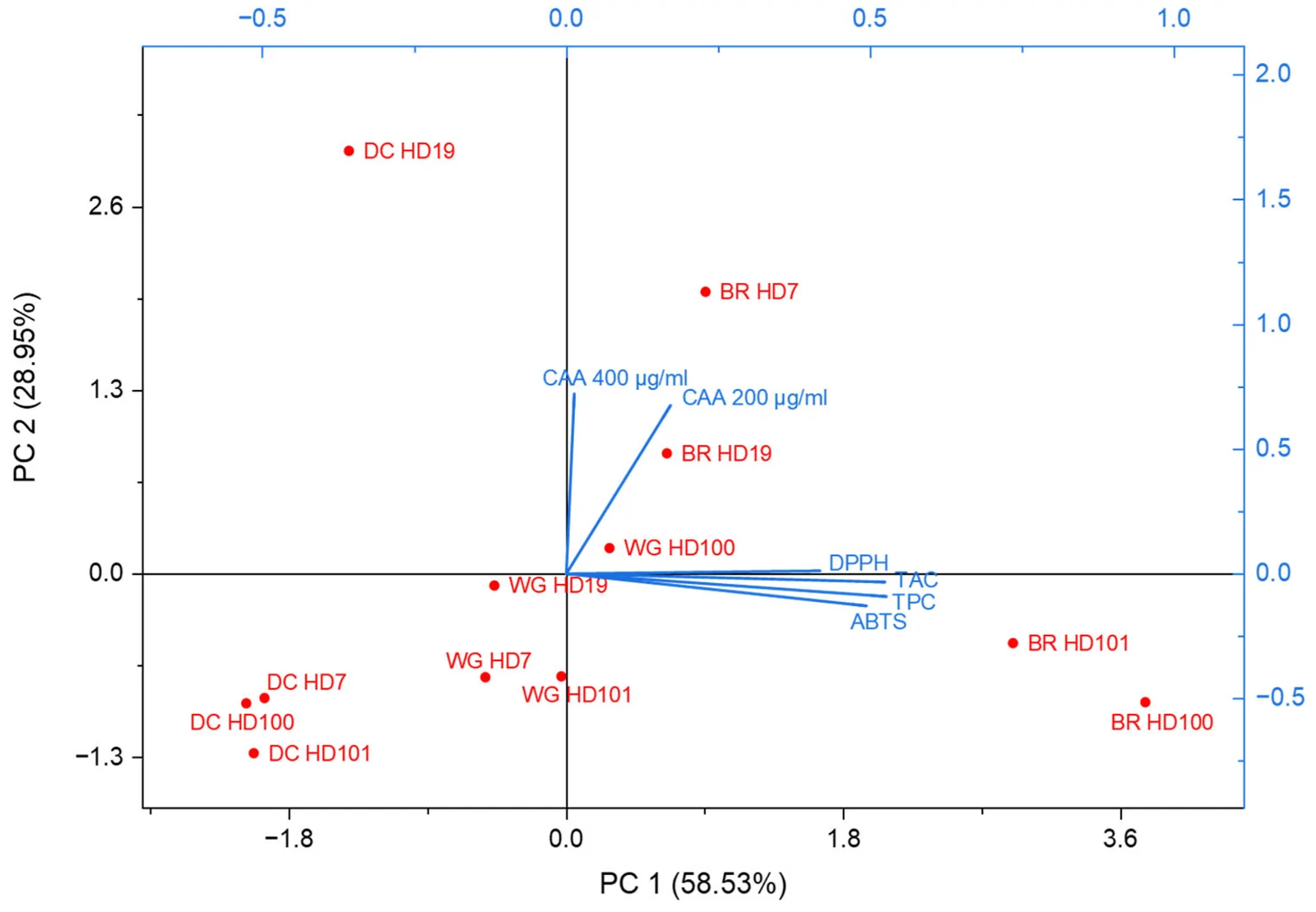
Principal component analysis (PCA) illustrating the clustering of the analyzed sorghum genotypes and their respective fractions based on measured variables.

PCA was performed to visualize interrelationships among genotypes, milling fractions, and processing states, and to correlate these with radical scavenging capacity and CAA. The first two principal components (PC1 and PC2) explained a total of 87.48% of the variance, with PC1 and PC2 accounting for 58.53% and 28.95%, respectively (Figure 5). This exceptionally high degree of explained variance indicates that the selected parameters, TPC, TAC, ABTS, DPPH, and CAA effectively discriminate the functional potential of these novel cold-adapted Dutch sorghum genotypes. Sorghum genotypes and milling fractions are represented by red dots, while the measured variables are represented by blue arrows. The blue axes (top and right) correspond to variable loadings, where the black axes (bottom and left) represent sample scores.

The first cluster, located in the lower-right quadrant (positive PC1, negative PC2 scores), was characterized by the cooked bran fractions BR HD100 and BR HD 101, which were strongly and positively associated with PC1 and closely aligned with the loadings for TAC, TPC, and ABTS. Their spatial positioning suggests that thermal processing in these pigmented genotypes optimizes the recovery of bioactive compounds, reaching the highest functional potential observed in this study. Furthermore, the strong alignment between the TPC and TAC vectors and the ABTS loading confirms that these phenolic compounds are the primary drivers of the high radical scavenging capacity in these specific bran fractions. A second cluster, located in the upper-right quadrant (PC1 positive, PC2 positive), comprised BR HD7, BR HD19, and WG HD100, which were closely associated with the positive loadings of CAA at 200 and 400 µg/mL on PC2, while DPPH exhibited a relatively low loading on this component.

In contrast, the remaining DC samples (DC HD7, HD100, and HD101) were clustered tightly in the lower-left quadrant (PC1 negative, PC2 negative), opposite to all phytochemical and antioxidant loadings. The tight clustering of these endosperm fractions, regardless of genetic background, demonstrates that milling fractionation is a more dominant driver of antioxidant potential than genotype alone. This statistical separation underscores the universal vulnerability of the endosperm to nutrient depletion during decortication and subsequent thermal processing, highlighting the critical role of the bran layer in maintaining the grain’s functional integrity. Whole-grain samples clustered near the origin and were therefore poorly discriminated by PC1 and PC2. These results may indicate that milling fraction contributed more to sample separation than genotype, which is consistent with the localization of phenolic compounds in the outer grain layers.

While PCA in this study effectively captured the complex relationships among genotype, milling fraction, processing state, and antioxidant properties, several limitations must be acknowledged. Only one cooking method (boiling) was evaluated, which may not represent the full range of effects produced by alternative processing techniques such as steaming, roasting, or extrusion. The identification of anthocyanins was based on retention time and UV spectra, without confirmation via authentic standards or mass spectrometry, which may limit compound specificity. Furthermore, antioxidant capacity was assessed using in vitro chemical and cell-based assays, which, although informative, may not fully predict in vivo bioavailability or physiological effects. Future research should address these limitations by expanding processing methods, employing advanced analytical confirmation for phytochemical identification, and including in vivo assessments of phenolic bioavailability and health impact.

## 4. Conclusions

This study demonstrates that cold-climate-bred Dusormil sorghum genotypes exhibit substantial variation in TPC and TAC across different grain types and milling fractions. The bran of HD100 exhibited the highest TPC (39.49 mg FAE/g), TAC (151.10 mg LUE/L) and ABTS (1175.49 mg TE/g). In contrast, the lowest TPC and TAC were observed in cooked decorticated HD7 (0.45 mg FAE/g) and HD101 (1.88 mg LUE/L), respectively. Cooking increased TPC in some fractions, particularly HD19 bran (approximately 64%), but reduced TAC by as much as 39% in HD100 bran.

Cooking exerted a dual effect: it increased TPC in certain sorghum fractions, with the greatest increase observed in HD19 bran (approximately 64%), while reducing TAC by 20–40% across most genotypes and fractions. The observations highlight the importance of optimizing thermal processing to maximize phytochemical retention. These findings underscore that both grain fraction and processing method critically influence the nutritional and functional properties of Dusormil sorghum.

Overall, the results indicate that bran-enriched and whole-grain Dusormil sorghum fractions exhibit greater antioxidant potential than decorticated fractions. Future research should focus on optimizing processing conditions and evaluating the bioavailability and in vivo effects of these bioactive compounds to better understand their potential applications in sorghum-based functional foods for temperate climates.

## Figures and Tables

**Table 1:** TPC and TAC of cooked and uncooked anatomical fractions of cold-climate-grown sorghum grains.

Samples	TPC (mg FAE/g)		TAC (mg LUE/L)	
	Uncooked	Cooked	Uncooked	Cooked
Whole grain				
HD7	5.52 ± 0.14 ^e^	7.14 ± 0.60 ^f^	22.80 ± 2.60 ^ef^	19.38 ± 1.27 ^cd^
HD19	5.65 ± 0.05 ^e^	7.13 ± 0.37 ^f^	26.01 ±1.21 ^e^	20.71 ± 1.21 ^c^
HD100	15.83 ± 0.23 ^c^	13.6 ± 0.18 d	39.74 ± 3.45 ^de^	27.55 ± 0.24 ^c^
HD101	9.59 ± 0.13 ^d^	11.65 ± 0.07 e	27.81 ± 1.82 ^e^	26.91 ± 1.75 ^c^
Decorticated				
HD7	1.45 ± 0.20 ^f^	0.45 ± 0.03 ^g^	2.52 ± 0.69 ^f^	6.02 ± 0.24 ^de^
HD19	1.35 ± 0.08 ^f^	0.52 ± 0.01 ^g^	2.19 ± 0.36 ^f^	6.35 ± 0.36 ^de^
HD100	2.59 ± 0.07 ^f^	0.79 ± 0.01 ^g^	3.95 ± 0.14 ^f^	1.92 ± 0.14 ^e^
HD101	1.55 ± 0.04 ^f^	0.83 ± 0.02 ^g^	3.72 ± 0.94 ^f^	1.88 ± 0.15 ^e^
Bran				
HD7	13.77 ± 0.33 ^c^	15.23 ± 1.17 ^c^	61.75 ± 3.70 ^c^	43.20 ± 3.39 ^b^
HD19	8.33 ± 0.08 ^d^	13.64 ± 0.12 ^cd^	53.67± 2.16 ^cd^	50.84 ± 3.09 ^b^
HD100	39.49 ± 1.44 ^a^	39.43 ± 0.96 ^a^	151.10 ± 14.97 ^a^	92.84 ± 8.18 ^a^
HD101	29.50 ± 2.05 ^b^	31.47 ± 0.85 ^b^	86.40 ± 7.71 ^b^	89.79 ± 7.25 ^a^

TPC: Total phenolic content, FAE: Ferulic acid equivalents, TAC: Total anthocyanin content, and LUE: Luteolinidin equivalents. Values represent the mean ± SD (*n* = 3). Values followed by different letters in a column represent significant differences at *p* < 0.05.

**Table 2:** Relative concentrations of identified HPLC peaks in uncooked and cooked Dusormil sorghum grain and their milled fractions.

Sample	Compounds		
	Luteolinidin	Apigeninidin	Peonidin
Whole grain			
Uncooked			
HD7	**	*	*
HD19	**	**	**
HD100	*	**	*
HD101	**	*	*
Cooked			
HD7	*	*	*
HD19	*	*	*
HD100	*	*	
HD101	*	**	*
Decorticated grain			
Uncooked			
HD7	*	*	*
HD19	*	*	*
HD100	*	*	*
HD101	**	*	*
Cooked			
HD7	*	*	*
HD19	*	*	*
HD100	*	*	
HD101	*	*	*
Bran			
Uncooked			
HD7	**	**	**
HD19	**	**	**
HD100	**	**	**
HD101	**	*	**
Cooked			
HD7	**	*	**
HD19	**	*	**
HD100	**	*	*
HD101	**	**	*

* = <100,000 AU, ** = <1,000,000 AU, AU = area under the curve.

**Table 3:** Antioxidant activities of sorghum grain fractions before and after cooking.

Sample	DPPH (mg TE/g)		ABTS (mg TE/g)	
	Uncooked	Cooked	Uncooked	Cooked
Whole grain				
HD7	166.42 ± 2.62 ^b^	167.02 ± 4.8 ^abcd^	90.80 ± 4.07 ^f^	63.95 ± 1.43 ^f^
HD19	161.82 ± 8.16 ^b^	155.32 ± 11.62 ^cd^	59.49 ± 2.09 ^fg^	59.32 ± 4.96 ^fg^
HD100	164.22 ± 4.83 ^b^	161.12 ± 9.2 ^bcd^	161.30 ± 11.72 ^d^	165.74 ± 5.35 ^d^
HD101	165.32 ± 4.60 ^b^	154.82 ± 6.04 ^d^	127.08 ± 3.29 ^e^	95.30 ± 6.36 ^hef^
Decorticated				
HD7	22.62 ± 1.74 ^c^	24.67 ± 0.53 ^e^	15.29 ± 1.78 ^h^	13.79 ± 1.08 ^h^
HD19	29.30 ± 0.98 ^c^	28.15 ± 0.38 ^e^	19.87 ± 1.86 ^h^	17.57 ± 1.50 ^h^
HD100	29.12 ± 1.03 ^c^	28.27 ± 0.23 ^e^	53.27 ± 3.21 ^g^	22.14 ± 2.01 ^gh^
HD101	28.92 ± 0.74 ^c^	28.37 ± 2.27 ^e^	33.64 ± 1.89 ^gh^	19.62 ± 2.27 ^gh^
Bran				
HD7	170.00 ± 6.68 ^ab^	166.81 ± 3.99 ^abcd^	301.74 ± 14.08 ^c^	208.41 ± 1.65 ^c^
HD19	164.20 ± 6.84 ^b^	171.41 ± 6.16 ^ab^	167.08 ± 7.23 ^d^	113.41 ± 5.56 ^e^
HD100	180.20 ± 1.31 ^a^	177.81 ± 0.46 ^a^	1175.49 ± 24.2 ^a^	845.81 ± 44.13 ^a^
HD101	173.30 ± 0.60 ^ab^	170.71 ± 0.62 ^abc^	637.71 ± 20.41 ^b^	617.35 ± 17.01 ^b^

Values represent the mean ± SD of three replicates. Letters within columns represent significant differences at *p* < 0.05. ABTS (2, 2′-Azino-Bis-3-Ethylbenzothiazoline-6-Sulfonic Acid assay), DPPH (2, 2-Diphenyl-1-picrylhydrazyl assay), and TE (Trolox equivalents).

## Data Availability

The original contributions presented in this study are included in the article. Further inquiries can be directed to the corresponding author.
